# A Common Variant Associated with Dyslexia Reduces Expression of the *KIAA0319* Gene

**DOI:** 10.1371/journal.pgen.1000436

**Published:** 2009-03-27

**Authors:** Megan Y. Dennis, Silvia Paracchini, Thomas S. Scerri, Ludmila Prokunina-Olsson, Julian C. Knight, Richard Wade-Martins, Penny Coggill, Stephan Beck, Eric D. Green, Anthony P. Monaco

**Affiliations:** 1 Wellcome Trust Centre for Human Genetics, University of Oxford, Oxford, United Kingdom; 2 Genome Technology Branch, National Human Genome Research Institute, National Institutes of Health, Bethesda, Maryland, United States of America; 3 Laboratory of Translational Genomics, Division of Cancer Epidemiology and Genetics, National Cancer Institute, National Institutes of Health, Gaithersburg, Maryland, United States of America; 4 Department of Physiology, Anatomy, and Genetics, University of Oxford, Oxford, United Kingdom; 5 Wellcome Trust Sanger Institute, Genome Campus, Hinxton, Cambridge, United Kingdom; 6 UCL Cancer Institute, University College London, London, United Kingdom; University of Illinois at Urbana-Champaign, United States of America

## Abstract

Numerous genetic association studies have implicated the *KIAA0319* gene on human chromosome 6p22 in dyslexia susceptibility. The causative variant(s) remains unknown but may modulate gene expression, given that (1) a dyslexia-associated haplotype has been implicated in the reduced expression of *KIAA0319*, and (2) the strongest association has been found for the region spanning exon 1 of *KIAA0319*. Here, we test the hypothesis that variant(s) responsible for reduced *KIAA0319* expression resides on the risk haplotype close to the gene's transcription start site. We identified seven single-nucleotide polymorphisms on the risk haplotype immediately upstream of *KIAA0319* and determined that three of these are strongly associated with multiple reading-related traits. Using luciferase-expressing constructs containing the *KIAA0319* upstream region, we characterized the minimal promoter and additional putative transcriptional regulator regions. This revealed that the minor allele of rs9461045, which shows the strongest association with dyslexia in our sample (max *p*-value = 0.0001), confers reduced luciferase expression in both neuronal and non-neuronal cell lines. Additionally, we found that the presence of this rs9461045 dyslexia-associated allele creates a nuclear protein-binding site, likely for the transcriptional silencer OCT-1. Knocking down *OCT-1* expression in the neuronal cell line SHSY5Y using an siRNA restores *KIAA0319* expression from the risk haplotype to nearly that seen from the non-risk haplotype. Our study thus pinpoints a common variant as altering the function of a dyslexia candidate gene and provides an illustrative example of the strategic approach needed to dissect the molecular basis of complex genetic traits.

## Author Summary

Dyslexia, or reading disability, is a common disorder caused by both genetic and environmental factors. Genetic studies have implicated a number of genes as candidates for playing a role in dyslexia. We functionally characterized one such gene (*KIAA0319*) to identify variant(s) that might affect gene expression and contribute to the disorder. We discovered a variant residing outside of the protein-coding region of *KIAA0319* that reduces expression of the gene. This variant creates a binding site for the transcription factor OCT-1. Previous studies have shown that OCT-1 binding to a specific DNA sequence upstream of a gene can reduce the expression of that gene. In this case, reduced *KIAA0319* expression could lead to improper development of regions of the brain involved in reading ability. This is the first study to identify a functional variant implicated in dyslexia. More broadly, our study illustrates the steps that can be utilized for identifying mutations causing other complex genetic disorders.

## Introduction

Dyslexia, or reading disability (RD), is a condition that affects an individual's ability to read and spell in the absence of any obvious sensory or neurological impairment and despite adequate intelligence and educational opportunity [1]. RD is one of the most common learning disabilities in school-aged children, with a prevalence ranging from 5% to 17.5% [2],[3]. Although the specific causes of the disorder have yet to be elucidated, it is generally accepted that RD has a strong genetic component [4],[5]. Family studies have estimated a high heritability of RD, reporting an incidence of about 40% in siblings of affected individuals [6],[7]; twin studies have shown a concordance rate of 68% in monozygotic twins versus 38% in dizygotic twins [8].

Numerous candidate genes have emerged from genetic association studies and the characterization of chromosomal translocations in individuals with RD, including *DYX1C1* on 15q21 [9]–[11], *ROBO1* on 3p12 [12], *DCDC2* [13],[14] and *KIAA0319* [15]–[19] on 6p22, and *MRPL19* and *C2ORF3* on 2p12 [20]. Several of these genes have been implicated in brain development [21]. In particular, RNAi-knockdown studies suggest that *DYX1C1* [22]–[24], *DCDC2* [13],[25], and *KIAA0319* [26] play a role in neuronal migration during the development of the rat cortex. Interestingly, altered neuronal migration has been implicated in RD based on the only post-mortem anatomical study conducted to date [27]; specifically, the brains of dyslexic individuals were found to have subtle structural anomalies consistent with defective neuronal migration.

**Figure 1:**
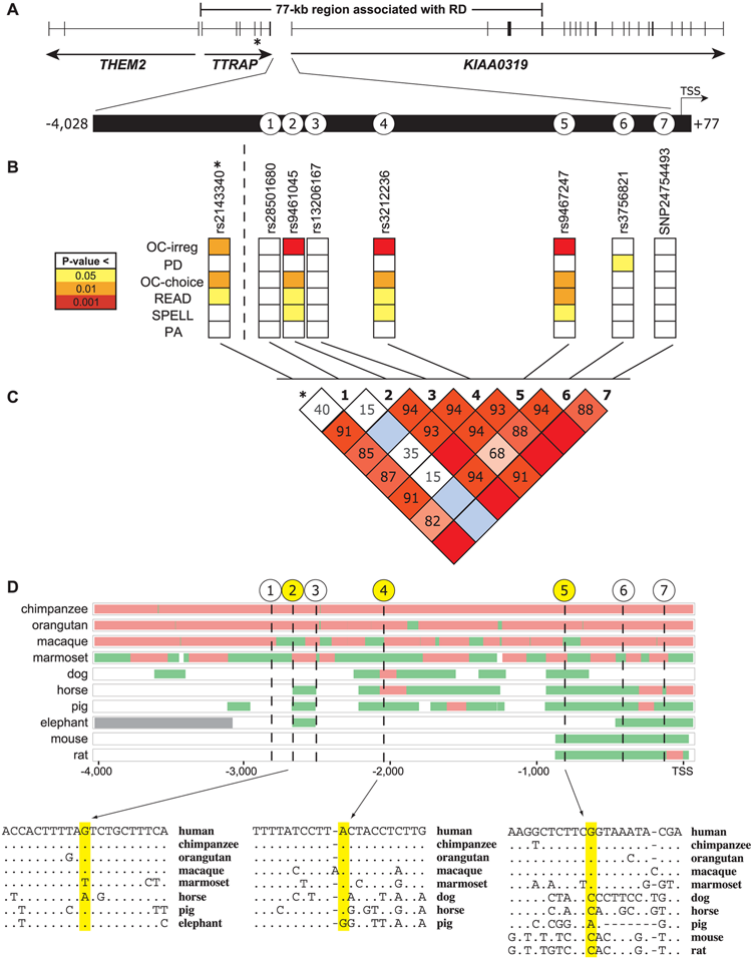
*KIAA0319* promoter region SNPs residing on the RD-associated risk haplotype. (A) Graphical representation of the location of the seven SNPs (corresponding to SNPs 1–7) on the RD-associated risk haplotype within the genomic region 4,028 bp upstream and 77 bp downstream of the *KIAA0319* TSS. The asterisk within *TTRAP* depicts the location of rs2143340, the risk haplotype-tagging SNP. (B) Associations of each SNP variant with the six indicated quantitative reading-related measures, as assessed by genotyping sample 1 (see Methods for details). (C) Representation of LD across the genomic region harboring these variants (calculated from the same genotyping data as in B), evaluated by Haploview version 4.0. The indicated numbers represent absolute D prime (D') between two loci. An empty red box represents complete LD, while an empty blue box indicates low LD. (D) MultiPip (percent identity plot) alignment of genomic sequence from the indicated vertebrate species across the region containing SNPs 1–7 compared to the human sequence derived from the non-risk BAC. Red indicates >75% identity between that species' sequence and the human sequence over 100 nucleotides; green indicates >50% identity between that species' sequence and the human sequence over 100 nucleotides; grey corresponds to sequence missing in that species; white corresponds to no sequence from that species aligning with the human sequence for the indicated interval. At the bottom, the nucleotide-level alignments are provided for the immediate regions encompassing SNPs 2, 4, and 5 (with the position of the SNP highlighted in each case; note that the depicted human sequence reflects the non-risk haplotype). A dot indicates that the corresponding base in that species matches the base in the human reference sequence.

We previously detected an RD-associated ‘risk haplotype’ through an association analysis of candidate genes residing at the chromosome 6p22 locus, which is one of the most consistently identified candidate regions by linkage studies [15]. Single-nucleotide polymorphisms (SNPs) were selected within brain-expressed genes and used for subsequent genetic analyses of RD. A 77-kb region of high inter-marker linkage disequilibrium (LD) that includes the first four exons of *KIAA0319*, all of *TTRAP*, and the region immediately upstream of *THEM2* (Figure 1A) showed significant associations with RD. Three SNPs captured most of the genetic variation and described the most common haplotype in the 77-kb region. One of these haplotypes, which was effectively tagged by the rs2143340 marker, was found to be significantly associated with RD. The association between this risk haplotype and reading-related traits was detected in two independent family-based sample sets of U.K. and U.S. origin [15]. Association with the same region was reported in a completely independent study [16]. Most recently, we replicated the association between the risk haplotype and reading-related phenotypes in an unselected sample of more than 6,000 children from the Avon Longitudinal Study of Parents and Children (ALSPAC) [19]. Using a quantitative allele-specific gene expression assay, we showed that there is reduced expression of *KIAA0319* (but not *TTRAP* and *THEM2*) from the risk haplotype in both lymphoblastoid and neuronal cell lines [26]. These data are consistent with the findings of a comprehensive association study, which tested an identical set of SNPs within the chromosome 6p22 locus in two independent U.K. sample sets [17]. The strongest association with RD was found with SNPs near the first exon of *KIAA0319* in both sample sets. Taken together, these data suggest that the risk haplotype might harbor a regulatory variant that alters *KIAA0319* transcription.

Here, we report additional genetic and functional characterization of the risk haplotype, specifically focusing on variants within the putative regulatory element(s) immediately upstream of *KIAA0319*. Our results implicate one variant as the likely cause of reduced *KIAA0319* expression from the risk haplotype. More broadly, these findings are relevant for further understanding the role of *KIAA0319* in RD and brain development as well as for establishing the role of non-coding mutations in complex genetic diseases.

## Methods

### Genomic Sequencing

Thirteen human bacterial artificial chromosomes (BACs) spanning the 77-kb RD-associated region were obtained either from Children's Hospital Oakland Resource Institute or the California Institute of Technology. The BACs were genotyped for the three risk haplotype-tagging SNPs (rs4504469, rs2038137, and rs2143340 [15]) using the Sequenom platform, according to the manufacturer's instructions. BACs RP11-195J19 [containing the risk haplotype (‘risk BAC’); GenBank accession number CR925830] and RP11-948M1 [containing a non-risk haplotype (‘non-risk BAC’); GenBank accession number CR942205] were chosen for their similar genomic coverage. Both BACs were sequenced at the Wellcome Trust Sanger Institute. Variants were detected by pair-wise comparisons using AlignX in the Vector NTI Advance 9 program (Invitrogen).

### Association Analysis

The collection of families used for quantitative trait association has been extensively described [15]. Briefly, all probands and siblings from our complete Oxford set of 264 unrelated nuclear families were identified from the dyslexia clinic at the Royal Berkshire Hospital (Reading, U.K.) and were administered a battery of psychometric tests. The following reading-related measures were used for statistical analyses: orthographic coding using irregular words (OC-irreg), phonological decoding ability (PD), orthographic coding assessed by forced word choice test (OC-choice), single-word reading ability (READ), spelling ability (SPELL), phonemic awareness (PA), and measures of IQ [verbal (SIM) and nonverbal (MAT)]. The scores were adjusted for age and IQ, and then standardized against a normative control data set as described [28],[29].

SNP genotyping was performed using either the MassARRAY hME or iPLEX system (Sequenom), according to the manufacturer's instructions (all primer sequences are available upon request). Marker-trait association was evaluated using the “total” association model with the QTDT package [30]. Variants were initially tested for association with the reading-related traits in a sample set consisting of 89 U.K. families previously described by Francks et al. [15], referred to as ‘sample 1.’ The LD among SNPs in this sample was determined using Haploview version 4.0 (http://www.broad.mit.edu/mpg/haploview) [31]. SNPs showing significant associations were tested in the entire sample of 264 families, referred to as ‘entire U.K. set,’ as well as in a phenotypically severe sample subset consisting of 126 families described previously [15],[17] and referred to as ‘severe U.K. subset.’ Briefly, the severe U.K. subset was chosen based on scores >0.5 SD below a composite mean score of the PD and OC-irreg traits, the two measures that contribute to the greatest degree to the chromosome 6p22 linkage peak [15].

### Multi-Species Sequence Comparisons

Genomic sequences orthologous to the interval between *TTRAP* and *KIAA0319* were obtained from publicly available databases (http://genome.ucsc.edu for chimpanzee, orangutan, macaque, marmoset, dog, mouse, and rat; http://www.ncbi.nlm.nih.gov/blast/Blast.cgi for horse, pig, and elephant). A multi-sequence alignment of these sequences was generated with MultiPipMaker (http://pipmaker.bx.psu.edu/pipmaker) using the sequence of the non-risk BAC as the human reference [32].

### Haplotype-Specific *KIAA0319* Promoter Region Constructs

The genomic segment immediately upstream of *KIAA0319* [−4,028 bp to +77 bp relative to the transcription start site (TSS)] from the non-risk BAC was cloned into the luciferase-expressing pGL3-Basic vector (Promega) using BAC recombineering [33]. Specifically, the pGL3-Basic vector was linearized with the restriction enzyme KpnI (New England Biolabs) and gel-purified (Qiagen). PCR amplification (Bioxact Long, Bioline) was performed using the linearized pGL3-Basic vector as the template and appropriate recombineering primers (see Text S1 for sequences). Electrocompetent cells containing the non-risk BAC were generated as described [34]. Column-purified (Qiagen) PCR product (2 µg), consisting of linearized pGL3-Basic vector flanked by homologous sequence to the non-risk BAC, was electroporated into 25 µl of temperature-induced SW102 *E. coli* [34] containing the non-risk BAC, and the cells were plated onto LB agar containing 100 µg/ml ampicillin and incubated at 32°C for 30 hours.

Constructs harboring various deletions were engineered by removing the segment between the restriction sites for EcoRV (−4,026 bp from the TSS) and the following: PmlI (−2,802 bp), BstXI (−2,185 bp), NsiI (−1,728 bp), PvuII (−940 bp), StuI (−544 bp), Bpu10I (−216 bp), Bsu36I (−97 bp), and BssHII (−24 bp). Site-directed mutagenesis of the full-length construct was performed using the QuikChange XL Site-Directed Mutagenesis kit (Stratagene) according to the manufacturer's protocol (primer sequences used for the mutagenesis are provided in Text S1). All mutated constructs were sequenced to ensure the absence of unwanted additional mutations.

### Luciferase Assays

SHSY5Y, SK-N-MC, and HEK293T cell lines were grown according to ECACC guidelines at 37°C with 5% CO_2_. All three cell lines were grown in 96-well plates at a concentration of 2.4×10^4^ cells/well for SHSY5Y, 3.5×10^4^ cells/well for SK-N-MC, and 1.5×10^4^ cells/well for HEK293T. After 24 hours, the cells were co-transfected with 0.05 pmol of the pGL3-derived construct (e.g., containing the non-risk haplotype, deletions, or mutations; note that a promoter-less pGL3-Basic construct was used as a negative control) and 2 ng of pRL-CMV with 20 µl Lipofectamine 2000 (Invitrogen). At 3–4 hours post-transfection, the medium was replaced. At 48 hours post-transfection when the cells had reached approximately 90% confluency, cells were lysed, and the luminescence was assayed using the Dual Luciferase Reporter Assay (Promega). The luminescence of 20 µl of lysis product was measured using a microplate luminometer (Luminoskan Ascent, Thermo Fisher Scientific).

The transfection efficiencies were normalized to the level of pRL-CMV renilla luciferase activity, and the results reflected as ‘relative luciferase activity’ (RLA). The RLA for each transfection were scaled so that the pGL3-Basic construct (in the case of constructs harboring deletions) or the full-length non-risk haplotype-containing construct (in the case of mutagenized constructs) yielded a 1.0 RLA. All transfections were performed in quadruplicate and repeated at least three times (twelve biological replicates in total). An unpaired two-sided t-test was used to compare the RLAs between the non-risk haplotype and mutagenized constructs.

### Electrophoretic Mobility Shift Assays (EMSA)

To create double-stranded EMSA probes carrying risk and non-risk alleles of the RD–associated SNPs, complementary oligonucleotides (see Text S1 for sequences) were annealed, end-labeled with [γ-^32^P]ATP (PerkinElmer) using 10 units of T4 polynucleotide kinase (Promega), and column-purified (GE Healthcare). Equal amounts of nuclear extract from the SHSY5Y cell line, prepared using a nuclear extraction kit (Cayman Chemical), were pre-incubated with or without an unlabeled double-stranded ‘competitor’ DNA in the presence of DNA-binding buffer (Promega) for 10 minutes at room temperature, and then incubated with the relevant ^32^P-labeled probe (17.5 fmol/sample) for 20 minutes at room temperature. For the ‘supershift EMSA’ [35], 2 µg of appropriate EMSA-grade concentrated antibody [OCT-1 (octamer-1), sc-232x and CRX (cone-rod homeobox), sc-30150x; Santa Cruz Biotechnology] was then added, and the sample was incubated at 4°C overnight. DNA-protein complexes were electrophoretically separated on a 6% polyacrylamide 0.5× TBE DNA retardation gel (Invitrogen) at room temperature, dried at 80°C for 1 hour, and visualized using a Fujifilm FLA-5000 image analyzer.

### RNA Silencing Studies

SHSY5Y cells, chosen because of their heterozygosity for the RD-associated risk haplotype, were reverse-transfected with corresponding siRNA cocktails or with Lipofectamine only (‘mock-transfected’). Briefly, SHSY5Y cells (4×10^5^ cells/well) were plated in 24-well plates just before transfection and mixed with pre-incubated siRNA cocktails. For the cocktails, siRNAs for OCT-1 (sc-36119, Santa Cruz Biotechnology), a positive control [GAPDH (glyceraldehyde 3-phosphate dehydrogenase), AM4605, Ambion], or a scrambled negative control (AM4636, Ambion) were diluted with Opti-MEM media and pre-incubated with Lipofectamine 2000 (Invitrogen). Two concentrations of siRNA were used for all experiments: 1.5 and 3.0 µM. The results were consistent with both concentrations, but there was less variation and the results were more statistically significant with the 1.5 µM concentration. After incubation at 37°C with 5% CO_2_ for 24 hours, the medium was replaced, and the cells were incubated for an additional 24 hours. All siRNA transfections were performed in 6 biological replicates for each concentration of siRNA. Subsequently, total RNA from the cells was prepared with Trizol reagent (Invitrogen) and the RNeasy miniprep kit (Qiagen). cDNA was synthesized from 1 µg of total RNA using the Superscript III First Strand Reverse Transcriptase Kit and random hexamers (Invitrogen).

Effects on gene expression by OCT-1 and GAPDH siRNAs, compared to scrambled siRNA, were evaluated by quantitative real-time PCR (qRT-PCR) with TaqMan expression assays (4333764F for GAPDH and HS00231250_m1 for OCT-1, Applied Biosystems). Expression was measured in siRNA-transfected and mock-transfected samples, and normalized to the level of expression of endogenous B2M (ß2-microglobulin), which is not affected by siRNA transfection (assay HS00187842_m1, Applied Biosystems). For each sample, expression was measured in 4 technical replicates, and average values were used for analysis.

Allele-specific expression in cDNA samples from different transfections was measured in quadruplicate by use of allele-discriminating TaqMan genotyping assays for SNPs rs807541 and rs4504469 (C___3073667_1_ and C___390135_10, respectively; Applied Biosystems). Both SNPs are located within coding sequence of *KIAA0319*, and therefore both alleles could be detected in cDNA. The alleles of these SNPs represent the risk and non-risk haplotypes: the risk haplotype allele of rs4504469 was established previously [26], while the risk haplotype allele of rs807541 was established by sequencing cloned cDNA derived from SHSY5Y cells. For each assay, a standard curve consisting of 10 dilutions of two HapMap DNA samples homozygous for either the risk or non-risk haplotype allele was generated (rs4504469: NA10847, NA12761; rs807541: NA10847, NA18858). The standard curve was used to validate the assay quality and to generate a regression equation necessary for determining the relative allelic ratio in the experimental samples. The relative ratio of the two alleles (A and B) was measured as the ratio between VIC and FAM fluorofores, which were attached to the two different corresponding allele-specific probes in each case. Specifically, the C_t_ (cycle at threshold) values were averaged between technical replicates, and the differences between the two alleles were calculated as ratio(A/B) = ratio(VIC/FAM) = C_t_(VIC)−C_t_(FAM) = dC_t_. The ratios of known dilutions of the HapMap DNA samples were plotted relative to dC_t_, and a linear regression model fitted to the data. The allelic ratios for the experimental samples were calculated using dC_t_ in the regression equation. An unpaired two-sided t-test was used to compare the means between groups of samples.

## Results

### Identification and Analysis of Sequence Variants on the Risk Haplotype

Pair-wise sequence comparison of the risk and non-risk BAC sequences revealed eight variants within the 4-kb region between *TTRAP* and *KIAA0319*: one simple repeat and seven SNPs [designated SNP 1 through SNP 7 (Figure 1A)]. These seven *KIAA0319* promoter region SNPs were genotyped in sample 1 (see Methods) and tested for association with various reading-related traits. In addition to the previously reported associations with rs3212236 (SNP 4) [17] and rs9467247 (SNP 5) [15], we found that rs9461045 (SNP 2) is significantly associated with many reading-related traits (Figure 1B and Table S1). Specifically, the minor alleles of these SNPs are most significantly associated with OC-irreg (P = 0.0002, SNP 2; P = 0.0002, SNP 4; P = 0.0001, SNP 5). An evaluation of LD across the region (Figure 1C) showed that all SNPs but rs28501680 (SNP 1) are in strong LD with rs2143340, the previously implicated risk haplotype-tagging SNP (residing within the *TTRAP* gene) [15]. SNPs 2 and 5 are in perfect LD with each other, sharing the same minor allele frequency of 0.19 (i.e., the minor alleles of both SNPs always occur together), with the slight differences in association P-values likely reflecting different genotyping success rates (Table S2). We followed up these findings by genotyping SNPs 2, 4, and 5 in both the entire U.K. set (Table 1 and Table S3) and the severe U.K. subset (Table 1 and Table S4). SNPs 2 and 5 show the strongest association detected so far with these samples. Both SNPs are most significantly associated with OC-irreg (P = 0.0046, SNP 2; P = 0.0025, SNP 5) in the entire U.K. set. Additional significant associations were found with the severe U.K. subset for OC-irreg (P = 0.0006, SNP 2; P = 0.0003, SNP 5), OC-choice (P = 0.0003, SNP 2; P = 0.0001, SNP 5), and READ (P = 0.0003, SNP 2; P = 0.0002, SNP 5).

**Table 1:** Genetic associations for markers genotyped in selected U.K. sample sets.

SNP	P-value for Trait^a^									
	Entire U.K. set (630 Siblings, 264 Families)					Severe U.K. subset (313 Siblings, 126 Families)				
	OC-irreg	PD	OC-choice	READ	SPELL	OC-irreg	PD	OC-choice	READ	SPELL
rs9461045 (SNP 2)	0.0046		0.0097		0.0104	0.0006	0.0489	0.0003	0.0003	0.0018
rs3212236 (SNP 4)	0.0175		0.018		0.0209	0.0013		0.0006	0.0008	0.0024
rs9467247 (SNP 5)	0.0025		0.0044		0.0084	0.0003	0.0362	0.0001	0.0002	0.0020

a Uncorrected for multiple testing with only significant P-values<0.05 shown. All associations with low reading scores are with the minor alleles of the SNPs. None of the SNPs were associated with the PA trait in either sample set.

We also performed comparative analyses of the genomic region 4 kb upstream of the *KIAA0319* TSS using sequences from 11 vertebrate species (Figure 1D). Multi-species sequence comparisons can reveal genomic segments under evolutionary constraint due to their functional importance [36]–[39], such as serving a role in transcriptional regulation. Overall, there is little conservation of this upstream region across species, evident by a paucity of multi-species conserved sequences identified on the UCSC Genome Browser (http://genome.ucsc.edu) [40] ‘Most Conserved’ track (Figure S1), which compares orthologous sequences from 12 different species. The most pronounced conservation across species extends from the TSS to approximately 1 kb upstream of *KIAA0319*. This region includes SNPs 5, 6, and 7, and likely encompasses the promoter and perhaps other upstream elements important in regulating *KIAA0319* expression. Examination of this region using the UCSC Genome Browser reveals a predicted CpG island, a DNase I hypersensitive site, a FirstEF-predicted promoter, and evidence for sequence conservation in certain species (Figure S1). SNP 5 is the only variant within this conserved region showing association with RD; analysis of SNP 5 reveals that the nucleotide on the non-risk haplotype (G allele) is conserved across primates only (Figure 1D). In the case of the other two associated variants (SNPs 2 and 4), the SNP 2 nucleotide on the non-risk haplotype (G allele) is conserved across all species examined except marmoset and horse, while the SNP 4 nucleotide on the non-risk haplotype (A allele) is conserved across all species examined except pig. Note that the sequences encompassing SNPs 2 and 4 could not be aligned with orthologous mouse or rat sequences.

### Characterization of the Putative *KIAA0319* Promoter Region

**Figure 2:**
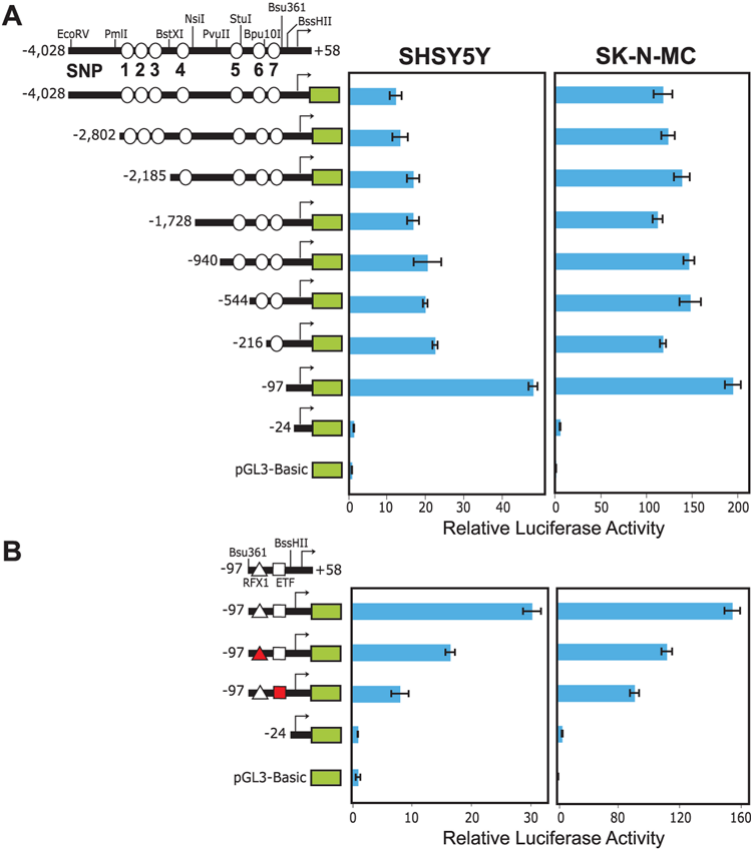
Luciferase-based expression analysis of the putative *KIAA0319* promoter region. (A) Luciferase-expressing constructs containing different portions of the *KIAA0319* promoter region from the non-risk haplotype were generated. Restriction sites relevant to the creation of the depicted “deletion series” of constructs are shown, as are the locations of SNPs 1–7 (see Figure 1A). Each construct was transfected into SHSY5Y and SK-N-MC neuronal cell lines and subsequent luciferase expression measured; all assays were performed in quadruplicate and repeated at least three times. RLA for each construct was scaled such that pGL3-Basic activity equaled 1.0. Error bars represent the standard error of the mean. The green box in each construct represents the proximal end of *KIAA0319*, with the arrow indicating the TSS. (B) Additional studies were performed with constructs containing disrupted RFX1- or ETF-binding sites (represented by a red triangle and red square, respectively).

We generated a series of luciferase-expressing constructs containing progressively smaller segments of the genomic region immediately upstream of *KIAA0319* (derived from the non-risk BAC), and tested each construct in two neuronal cell lines, SHSY5Y and SK-N-MC (Figure 2A). Neuronal cell lines were chosen based on the strong expression of *KIAA0319* in the developing human brain [26]. These studies indicated the presence of promoter activity between −24 and −97 bp of the *KIAA0319* TSS. TRANSFAC [41] analysis of this interval revealed predicted binding sites for the transcription factors RFX1 (regulatory factor X, 1) and ETF (epidermal growth factor receptor transcription factor); we also identified the same RFX1-binding site using the UCSC Human Genome Browser (Figure S1). Site-directed mutagenesis of the RFX1- or ETF-binding site significantly reduced luciferase expression (Figure 2B), although neither mutated site was associated with a complete loss of promoter activity. Interestingly, ETF is known to drive transcription from promoters that are GC-rich and lack a TATA box [42]; this is the case for the putative promoter of *KIAA0319*, which includes an *in silico*-predicted CpG island (Figure S1). None of the seven SNPs we identified between *TTRAP* and *KIAA0319* reside in this putative promoter region.

Additionally, transcriptional silencing activity appeared to be associated with the region from −97 to −216 bp of the *KIAA0319* TSS, an interval in which TRANSFAC predicted a Pax-6 (paired box gene 6) binding site. While SNP 7 falls within this region, it does not interrupt this predicted binding site or show strong association with any reading-related traits.

### Influence of Risk Variants on Gene Expression and Nuclear Protein Binding

**Figure 3:**
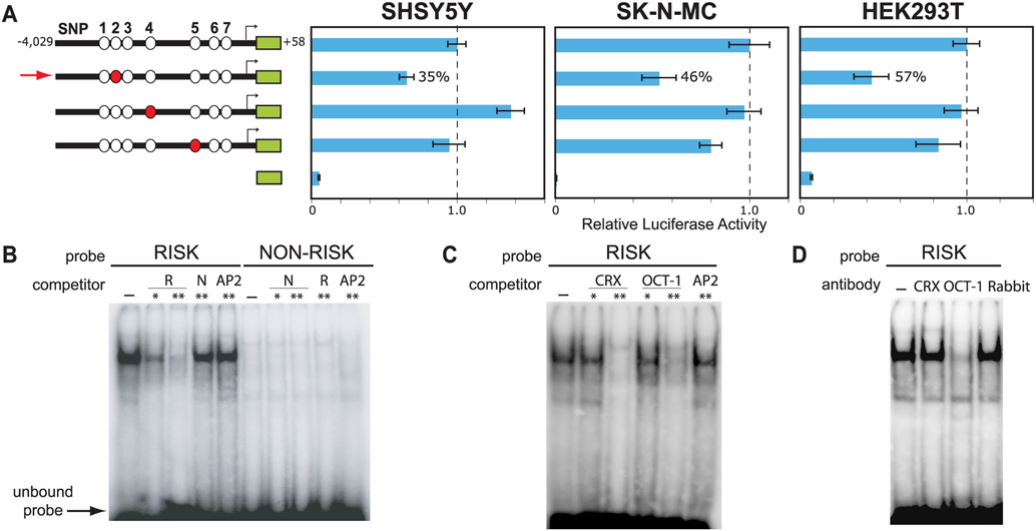
Effect of risk versus non-risk variants on luciferase expression and nuclear protein binding. (A) Luciferase expression from constructs containing the risk versus non-risk variants of SNPs 2, 4, and 5 was measured in SHSY5Y, SK-N-MC, and HEK293T cells. White and red circles represent the non-risk and risk variants, respectively (see Figure 2 for additional features of the depicted constructs). All assays were performed in quadruplicate and repeated at least three times. Error bars represent the standard error of the mean. The vertical dashed line represents the RLA measured for the construct containing the non-risk haplotype (set at 1.0 RLA); note that this reflects a different scale than the RLA depicted in Figure 2. For all three cell lines, only the construct containing the SNP 2 risk variant (denoted with a red arrow) yielded a significant RLA difference compared to the construct containing the non-risk haplotype, as analyzed using an unpaired two-sided t-test (P = 8.32×10^−10^, SHSY5Y; P = 3.92×10^−7^, SK-N-MC; P = 4.02×10^−8^, HEK293T). (B) EMSA testing the binding of SHSY5Y nuclear protein(s) to probes containing the SNP 2 risk versus non-risk variant. The presence of a competitor is denoted above each lane: -, no competitor; R, risk competitor; N, non-risk competitor; AP2, competitor containing an AP2-binding site (negative control); and *, 10-fold and **, 100-fold excess of competitor, respectively. (C) EMSA testing the binding of SHSY5Y nuclear protein(s) to probes containing the SNP 2 risk variant in the presence of competitors containing binding sites for CRX, OCT-1, and AP2 (negative control). (D) Supershift EMSA testing the binding of SHSY5Y nuclear protein(s) to probes containing the SNP 2 risk variant in the presence of anti-CRX or -OCT-1 antibody or general rabbit antiserum.

We next investigated the effect of the three variants highly associated with reading-related traits (SNPs 2, 4, and 5) on luciferase expression using mutagenized versions of the above-described non-risk haplotype construct (Figure 3A). In these studies, we directly compared the non-risk versus risk allele for each SNP, measuring luciferase expression in SHSY5Y and SK-N-MC cells as well as in HEK293T, a human embryonic kidney cell line; this allowed examination of promoter activity in neuronal as well as non-neuronal cell lines. Introduction of the SNP 2 risk variant significantly reduced luciferase expression (by 35–57%) in all three cell lines. The SNP 4 risk variant increased luciferase expression in SHSY5Y cells, but not in SK-N-MC or HEK293T cells; the SNP 5 risk variant had a negligible effect on luciferase expression in these cell lines. These findings suggest that SNP 2 may contribute to the reduced *KIAA0319* expression seen from the risk haplotype.

EMSAs were performed to investigate the potential role of SNP 2 in modulating transcription factor binding. A probe corresponding to the risk (but not the non-risk) allele of SNP 2 binds nuclear protein(s) in an EMSA (Figure 3B). No allele-specific nuclear protein binding was detected by EMSA for either SNP 4 or 5 (data not shown). *In silico* analysis of the sequence encompassing SNP 2 using TRANSFAC revealed that the risk variant creates a putative binding site for CRX and OCT-1. Accordingly, we performed an EMSA in the presence of unlabeled competitors containing known binding sites for human CRX and OCT-1, respectively. Both competitors ablated binding of the nuclear protein(s) to the probe containing the SNP 2 risk variant (Figure 3C). We also performed a supershift EMSA (see Methods) with anti-CRX or anti-OCT-1 polyclonal antibody, and found that the presence of the anti-OCT-1 (but not anti-CRX) antibody decreased the observed binding (Figure 3D). These data provide *in vitro* evidence of a functional mechanism by which the SNP 2 risk allele contributes to the reduced *KIAA0319* expression through creation of a binding site for the transcription factor OCT-1.

### Allele-Specific Effect of an OCT-1 Knock-Down on *KIAA031*9 Expression

**Figure 4:**
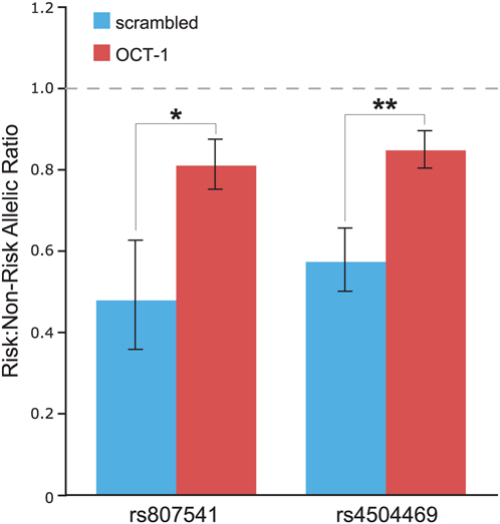
Effect of OCT-1 knock-down on *KIAA031*9 expression in SHSY5Y neuronal cells. *KIAA0319* expression from the risk versus non-risk haplotype measured in SHSY5Y neuronal cells transfected with a scrambled versus OCT-1–specific siRNA. Allele-specific *KIAA0319* expression of all samples was quantified by measurements of the allelic ratios of two heterozygous coding SNPs in *KIAA0319* (rs807541 and rs4504469). The results are presented as the global mean±the standard error of the mean of the measurements in the six biological replicates (*P = 0.004; **P = 0.0003). The horizontal dashed line at 1.0 represents equal *KIAA0319* expression from the risk and non-risk haplotypes.

Using an siRNA, we knocked-down OCT-1 expression in SHSY5Y cells, which Paracchini et al. [26] previously showed express *KIAA0319*, and are heterozygous for the risk haplotype. This siRNA reduced OCT-1 expression by about half (versus transfection with a scrambled siRNA, P = 0.0002). We then measured the effect of OCT-1 knock-down on *KIAA0319* expression using allele-specific qRT-PCR assays for two heterozygous coding SNPs residing within *KIAA0319* (rs807541 and rs4504469). These SNPs showed mean allelic ratios significantly lower than 1.0 (Figure 4) after transfection of a scrambled siRNA (negative control); in particular, the results for both SNPs indicate that *KIAA0319* expression from the risk haplotype is lower than from the non-risk haplotype, with risk:non-risk allelic ratios of X_rs807541_ = 0.48±0.13 and X_rs4504469_ = 0.57±0.08 (in agreement with values previously reported by Paracchini et al. [26]). Following OCT-1 knock-down, the allelic ratios were significantly closer to 1.0 (X_rs807541_ = 0.81±0.06 and X_rs4504469_ = 0.85±0.04), consistent with an increase in *KIAA0319* expression from the risk haplotype.

## Discussion

In this study, we sought to identify a variant(s) on the RD-associated risk haplotype [15] that decreases expression of *KIAA0319*. Our experimental data consistently indicate that the minor allele of rs9461045 (SNP 2) is likely to be functionally relevant for the development of RD. Specifically, we have shown that the risk allele of rs9461045: (1) is one of the markers most significantly associated with RD in our set of families; (2) decreases gene expression in luciferase-based assays; and (3) creates a binding site for a nuclear protein(s), likely to include the transcriptional silencer OCT-1. Moreover, the role of OCT-1 was further supported by the increase in *KIAA0319* expression from the risk haplotype upon siRNA-mediated knock-down of OCT-1.

The chromosome 6p22 risk haplotype is a well-established genetic risk factor for reading problems in populations of European descent, showing association in at least two sets of families with RD [15] and a large unselected set of additional individuals [19]. The data we present here help to provide an explanation for previous contradictory reports that failed to replicate an RD-association with the risk haplotype. Specifically, Luciano et al. [18] detected an opposite trend of association, showing that the same haplotype was associated with good (as opposed to poor) reading skills in an unselected Australian sample set. A different LD structure of the region in the population examined in this latter study can explain these apparently divergent findings, as previously suggested [19]. HapMap samples were analyzed for both markers, rs2143340 (the risk haplotype-tagging SNP) and rs9461045 (SNP 2), as shown in Figure S2; the detected LD differs among populations. LD is strong in the CEPH population (European descent), implying that haplotypes containing the minor allele of rs9461045 will also harbor the minor allele of rs2143340; LD between these two markers is not seen in three other HapMap populations. As such, the two markers will be present in all the possible haplotypes within these other populations, which makes it possible that, by chance, the minor allele of rs9461045 will appear more frequently in combination with the major allele of rs2143340. This scenario can explain why we see conflicting association results between studies using different populations, as is often the case in replication analyses of disease/trait associations [43]. This could certainly be the case for the Australian sample set, which is at least partially admixed. Thus, our study provides an empirical explanation for apparently contradictory complex trait-related genetic associations.

The precise function of *KIAA0319* has yet to be elucidated, but it appears to play a role in neuronal migration during brain development, similar to other RD candidate genes [44] and as evidenced by its specific pattern of expression in the developing human and mouse neocortex [26]. Additionally, *KIAA0319* is strongly expressed in human adult brain, specifically in the superior parietal cortex, primary visual cortex, and occipital cortex [13], areas thought to be important in reading [45]. Our studies identified two regions that may contribute to this expression specificity (Figure 2A). First, the *KIAA0319* promoter has a potential binding site for RFX1, a protein shown to regulate differentiation of ciliated sensory neurons in *C. elegans* [46] and *Drosophila* [47]. Second, the region implicated as a likely silencer element contains a predicted binding site for Pax-6, a transcription factor known to play a major role in regulating cortex development [48]. It is notable that the *Pax-6* and *KIAA0319* genes have similar expression patterns in the developing mouse and human brains [26], consistent with their potential transcriptional regulatory interactions.

The rs9461045 risk variant creates potential binding sites for CRX and OCT-1 transcription factors, although we could only find evidence for OCT-1 binding to the risk haplotype (Figure 3D). Both CRX and OCT-1 contain DNA-binding homeobox domains with similar recognition sites [49],[50]; it is thus possible that OCT-1 was able to bind both CRX and OCT-1 competitors, which would explain the observed ablation of risk probe-binding in the EMSA with either competitor (Figure 3C). OCT-1, also known as POU2f1 (POU domain, class 2, transcription factor 1), is a ubiquitously expressed member of the POU domain factor family [51]. This protein is involved in many biological processes, and has been shown to play a role in the formation of radial glia, the cells that provide a scaffold structure for neuronal migration [52]. OCT-1 can act as a transcriptional silencer by binding to an 8-bp AT-rich target (‘octamer’) near a promoter [53]. Notably, rs9461045 falls in a 120-bp AT-rich genomic region that has relatively higher sequence identity with the orthologous regions in the horse, pig, and elephant genomes compared to the surrounding region (Figure 1D). Further, it has been shown that such AT-rich regions are important for unzipping DNA during transcription [54] and are susceptible to binding by nuclear matrix attachment proteins, such as OCT-1 [53]. While the specific region encompassing rs9461045 is not highly conserved across mammals, recent findings suggest that upwards of 50% of authentic transcription factor-binding sites are not heavily conserved, at least not based on the methods used to date for identifying multi-species sequence conservation [55]. Since the rs9461045 risk variant appears to create a human-specific transcription factor-binding site that reduces gene expression, this site may not be under evolutionary constraint.

The studies reported here provide for the first time strong evidence implicating a specific variant to be functionally relevant for RD. Our findings provide new insights for understanding the role of *KIAA0319* in RD and brain development as well as for establishing the role of non-coding mutations in complex genetic diseases. A growing body of evidence suggests that variants residing in transcriptional regulatory elements (as opposed to coding regions) underlie many such disorders [56],[57]. Therefore, the experimental strategies described here more broadly illustrate a general approach that can be used for investigating the molecular basis of genetically complex diseases. Our findings also provide the first example, to our knowledge, of using siRNA to define the functional basis of allele-specific effects of genetic variants, and highlight the different approaches needed to implicate functional variants in complex genetic diseases.

## Supporting Information

Figure S1 Figure S1. UCSC Human Genome Browser snapshot (http://genome.ucsc.edu) using data from the Human May 2004 Assembly (chr6:24,753,365–24,758,893). Depicted is the region between *TTRAP* and *KIAA0319* showing (from top to bottom) the interval covered by the luciferase deletion series (see Figure 2A), the seven *KIAA0319* promoter region SNPs (see Figure 1A), *TTRAP* and *KIAA0319* genes, and various ‘Regulation and Comparative Genomics’ tracks.
(0.14 MB PPT)

Figure S2 Figure S2. LD structure of four HapMap populations at the *KIAA0319* locus. D' values are indicated, as represented through Haploview version 3.32. The black diamonds indicate LD values between rs2143340 (the risk haplotype-tagging SNP) and rs9461045 (SNP 2). Strong LD (red squares) between rs2143340 and SNP 2 is detected only in the population of European origin. Adapted from Paracchini et al. [19].
(0.40 MB PPT)

Table S1 Table S1. Marker-trait association P-values in sample 1 (89 families).
(0.06 MB PDF)

Table S2 Table S2. Genotype statistics for KIAA0319 promoter region SNPs.
(0.04 MB PDF)

Table S3 Table S3. Marker-trait association P-values in the entire U.K. set (264 families) - adapted from Harold et al. [17].
(0.07 MB PDF)

Table S4 Table S4. Marker-trait association P-values in the severe U.K. subset (126 families) - adapted from Harold et al. [17].
(0.07 MB PDF)

Text S1 Text S1. Supplementary methods.
(0.07 MB PDF)
