# Supplementary figures and images for: A Common Variant Associated with Dyslexia Reduces Expression of the KIAA0319 Gene
Source: PLoS Genet. 2009 Mar 27;5(3):e1000436. doi: 10.1371/journal.pgen.1000436 (PMC2653637; doi:10.1371/journal.pgen.1000436)

## Slide 1
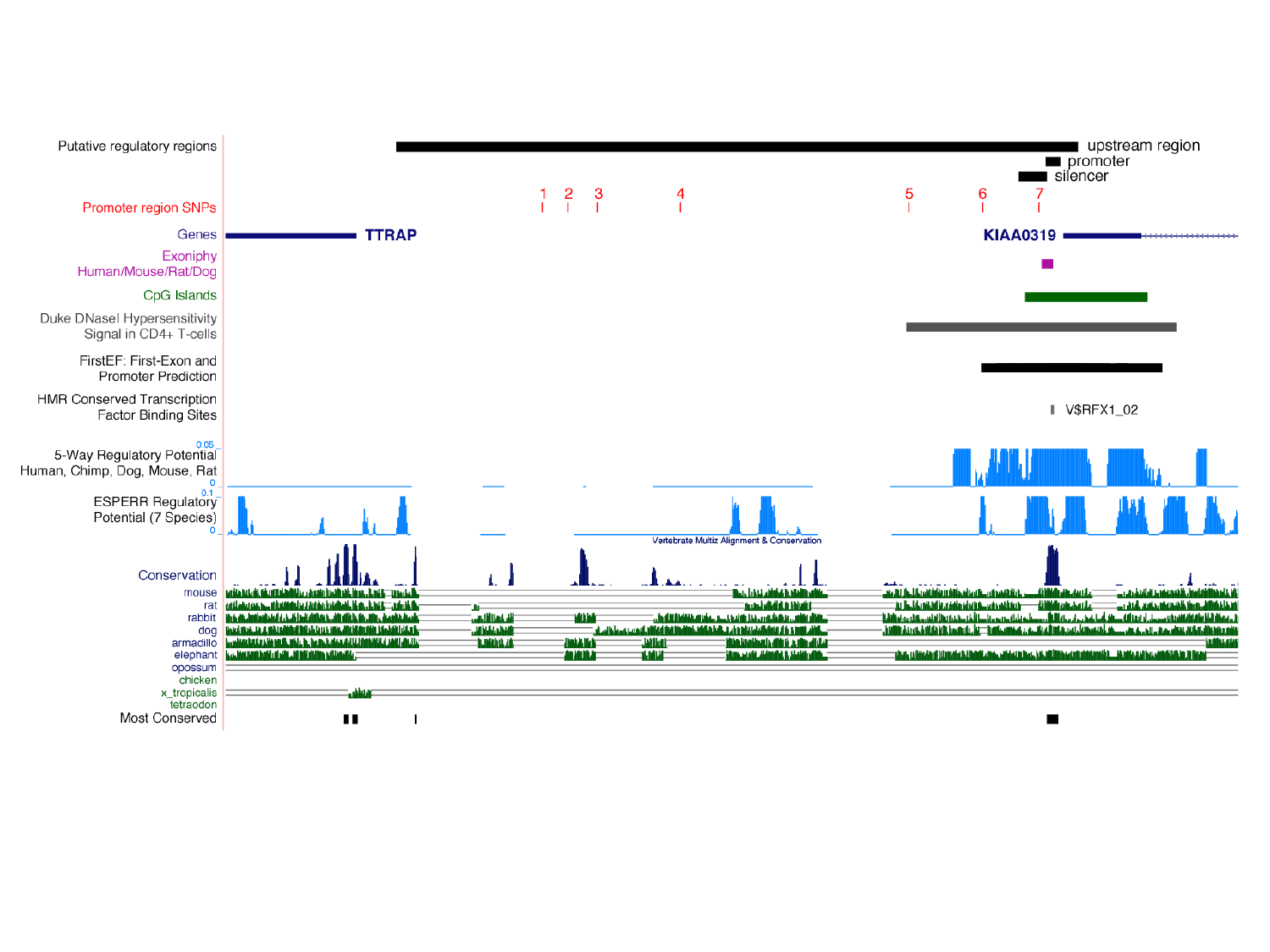

Supplement: Figure S1 — UCSC Human Genome Browser snapshot (http://genome.ucsc.edu) using data from the Human May 2004 Assembly (chr6:24,753,365–24,758,893). Depicted is the region between TTRAP and KIAA0319 showing (from top to bottom) the interval covered by the luciferase deletion series (see Figure 2A), the seven KIAA0319 promoter region SNPs (see Figure 1A), TTRAP and KIAA0319 genes, and various ‘Regulation and Comparative Genomics’ tracks. (0.14 MB PPT) [file pgen.1000436.s001.ppt]

## Slide 1
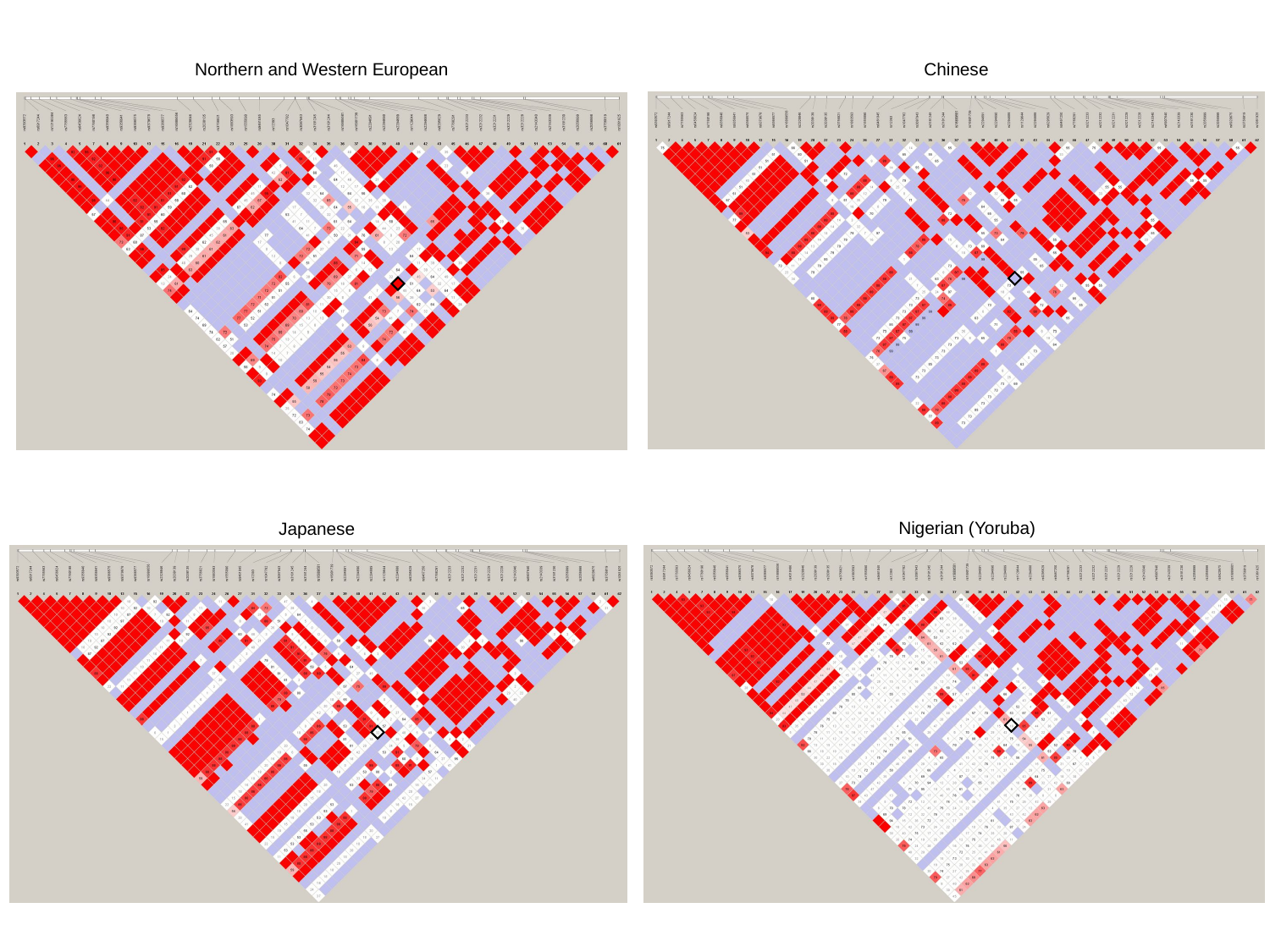

Chinese
Northern and Western European
Nigerian (Yoruba)
 Japanese

Supplement: Figure S2 — LD structure of four HapMap populations at the KIAA0319 locus. D' values are indicated, as represented through Haploview version 3.32. The black diamonds indicate LD values between rs2143340 (the risk haplotype-tagging SNP) and rs9461045 (SNP 2). Strong LD (red squares) between rs2143340 and SNP 2 is detected only in the population of European origin. Adapted from Paracchini et al. [19]. (0.40 MB PPT) [file pgen.1000436.s002.ppt]
