# Supplementary material for: A Common Variant Associated with Dyslexia Reduces Expression of the KIAA0319 Gene
Source: PLoS Genet. 2009 Mar 27;5(3):e1000436. doi: 10.1371/journal.pgen.1000436 (PMC2653637; doi:10.1371/journal.pgen.1000436)
Supplement: Table S1 — Marker-trait association P-values in sample 1 (89 families). (0.06 MB PDF) [file pgen.1000436.s003.pdf]

Table S1. Marker-trait association P-values in sample 1 (89 families)

Marker[1]	Gene	Risk Allele[2]	*P*-Value for Trait[3]					
			OC-irreg	PD	OC-choice	READ	SPELL	PA
rs1419228	*DCDC2*							
rs793862	*DCDC2*							
rs793834	*DCDC2*							
rs807701	*DCDC2*							
rs807724	*DCDC2*							
DCDC2_deletion	*DCDC2*	2	0.0298			0.0428		0.0478
rs7765678	*DCDC2*							
rs1087266	*DCDC2*							
rs4504469	*KIAA0319*	1	0.0227					0.0338
rs2179515	*KIAA0319*	1	0.0076					
rs730860	*KIAA0319*	2	0.0058		0.0042			
rs761101	*KIAA0319*	1	0.0173					
rs761100	*KIAA0319*	1	0.008					
rs2038137	*KIAA0319*	1	0.0262					
k_pr_del	Intergenic	1	0.0086					
**SNP24754493**	Intergenic							
**rs3756821**	Intergenic	2		0.0341				
rs9467247[4]	Intergenic	2	0.0001		0.0031	0.0085	0.0194	
rs1555090	Intergenic	1	0.0094			0.0803		
rs3212236[4]	Intergenic	2	0.0002		0.0023	0.0175	0.0491	
**rs13206167**	Intergenic							
**rs9461045**	Intergenic	2	0.0002		0.0049	0.013	0.0342	
**rs28501680**	Intergenic							
rs2143340[4,5]	*TTRAP*	2	0.0064		0.0052	0.0165		

[1] Markers new to this study are in bold
[2] 1 = major allele; 2 = minor allele
[3] Only nominally significant marker-trait associations (P < 0.05) are shown, and are uncorrected for multiple testing
[4] Re-genotyped in this study, resulting in different P-values than previously reported
[5] Risk haplotype-tagging SNP
