# Supplementary material for: A Common Variant Associated with Dyslexia Reduces Expression of the KIAA0319 Gene
Source: PLoS Genet. 2009 Mar 27;5(3):e1000436. doi: 10.1371/journal.pgen.1000436 (PMC2653637; doi:10.1371/journal.pgen.1000436)
Supplement: Table S2 — Genotype statistics for KIAA0319 promoter region SNPs. (0.04 MB PDF) [file pgen.1000436.s004.pdf]

Table S2. Genotype statistics for *KIAA0319* promoter region SNPs

Marker	SNP	Sample 1 89 families		Entire U.K. set 264 families		Severe U.K. subset 126 families	
		MAF[a]	GSR[b]	MAF	GSR	MAF	GSR
rs2143340	*	0.16	98.3	0.14	90.8	0.12	90.3
rs28501680	1	0.005	96.8	0.002	97.8	0	97.7
rs9461045	2	0.19	95.8	0.17	97.3	0.15	97.2
rs13206167	3	0.36	78.1	0.40	84.4	0.42	83.4
rs3212236	4	0.20	82.6	0.17	91.2	0.14	93.1
rs9467247	5	0.19	97.0	0.17	97.9	0.15	98.1
rs3756821	6	0.39	94.8	0.40	89.4	0.42	88.5
SNP24754493	7	0.14	91.5	N/A	N/A	N/A	N/A

[a]Minor allele frequency
[b]Genotype success rate (%)
*Risk haplotype-tagging SNP
