# Supplementary material for: A Common Variant Associated with Dyslexia Reduces Expression of the KIAA0319 Gene
Source: PLoS Genet. 2009 Mar 27;5(3):e1000436. doi: 10.1371/journal.pgen.1000436 (PMC2653637; doi:10.1371/journal.pgen.1000436)
Supplement: Table S3 — Marker-trait association P-values in the entire U.K. set (264 families) - adapted from Harold et al. [17]. (0.07 MB PDF) [file pgen.1000436.s005.pdf]

Table S3. Marker-trait association P-values in the entire U.K. set (264 families) – Adapted from Harold et al. [17]

Marker[1]	Gene	Risk Allele[2]	*P*-Value for Trait[3]					
			OC-irreg	PD	OC-choice	READ	SPELL	PA
rs1419228	*DCDC2*							
rs7762605	*DCDC2*							
rs793862	*DCDC2*	1		0.0424		0.0396	0.0226	
rs793834	*DCDC2*							
rs807701	*DCDC2*							
rs807726	*DCDC2*							
rs807724	*DCDC2*	1				0.0137	0.0115	0.0387
rs10806987	*DCDC2*							
DCDC2_deletion	*DCDC2*							
rs7765678	*DCDC2*							
rs793704	*DCDC2*							
rs1087266	*DCDC2*	1		0.0257				0.0053
rs2817220	*ALDH5A1*	1		0.0462				0.0226
rs2252525	*ALDH5A1*							
rs807517	*ALDH5A1*	1						0.0155
rs1054899	*ALDH5A1*	1					0.0204	
rs2760184	Intergenic							
rs807527	*KIAA0319*							
rs699463	*KIAA0319*	1	0.0412			0.0358	0.0101	0.0124
rs807530	*KIAA0319*							
rs807535	*KIAA0319*							
rs807540	*KIAA0319*							
rs807545	*KIAA0319*							
rs2817191	*KIAA0319*							
rs807521	*KIAA0319*	1						0.0485
rs807525	*KIAA0319*							
rs807507	*KIAA0319*							
rs807508	*KIAA0319*	1					0.0188	0.021

rs4504469	*KIAA0319*	1	0.0337				
rs4576240	*KIAA0319*						
rs4472344	*KIAA0319*						
rs16889511	*KIAA0319*						
rs4236032	*KIAA0319*	1	0.023			0.0051	0.003
rs9356939	*KIAA0319*	2	0.0434				
SNP24721800	*KIAA0319*	2	0.0464			0.0459	
rs9393567	*KIAA0319*	2	0.0443				
rs16889523	*KIAA0319*						
rs2745333	*KIAA0319*	1	0.014				
rs12194307	*KIAA0319*						
rs2179515	*KIAA0319*	1	0.0246				
rs730860	*KIAA0319*	2	0.0074		0.0042		
rs761101	*KIAA0319*	1	0.0265			0.0473	
rs761100	*KIAA0319*						
rs7766230	*KIAA0319*	2	0.0333		0.0138	0.0083	0.0106
rs6935076	*KIAA0319*						
rs6456624	*KIAA0319*	1	0.0167			0.0272	
rs2328846	*KIAA0319*	1	0.0257				
rs17491230[4]	*KIAA0319*	2	0.0031		0.0426	0.0213	0.0044
rs2038137	*KIAA0319*	1	0.035				
k_pr_del	Intergenic	1	0.0261				
**rs3756821**	Intergenic						
rs9467247[5]	Intergenic	2	0.0025		0.0044		0.0084
rs1555090	Intergenic	1	0.0198	0.0497		0.033	
rs3212236[5]	Intergenic	2	0.0175		0.018		0.0209
**rs13206167**	Intergenic						
**rs9461045**	Intergenic	2	0.0046		0.0097		0.0104
**rs28501680**	Intergenic						
rs3033236	*TTRAP*	2	0.0251		0.0205		
rs3212232	*TTRAP*						
rs2143340[5,6]	*TTRAP*	2	0.0367		0.0174		

[1] Markers new to this study are in bold
[2] 1 = major allele; 2 = minor allele
[3] Only nominally significant marker-trait associations ($P < 0.05$) are shown, and are uncorrected for multiple testing
[4] Equivalent to rs2235676 in Francks et al. [15]
[5] Re-genotyped in this study, resulting in different P-values than previously reported
[6] Risk haplotype-tagging SNP
