# Supplementary material for: A Common Variant Associated with Dyslexia Reduces Expression of the KIAA0319 Gene
Source: PLoS Genet. 2009 Mar 27;5(3):e1000436. doi: 10.1371/journal.pgen.1000436 (PMC2653637; doi:10.1371/journal.pgen.1000436)
Supplement: Table S4 — Marker-trait association P-values in the severe U.K. subset (126 families) - adapted from Harold et al. [17]. (0.07 MB PDF) [file pgen.1000436.s006.pdf]

Table S4. Marker-trait association P-values in the severe U.K. subset (126 families) – Adapted from Harold et al. [17]

Marker[1]	Gene	Risk Allele[2]	*P*-Value for Trait[3]					
			OC-irreg	PD	OC-choice	READ	SPELL	PA
rs1419228	*DCDC2*							
rs7762605	*DCDC2*							
rs793862	*DCDC2*							
rs793834	*DCDC2*							
rs807701	*DCDC2*							
rs807726	*DCDC2*							
rs807724	*DCDC2*							
rs10806987	*DCDC2*							
DCDC2_deletion	*DCDC2*	2	0.0298			0.0428		0.0478
rs7765678	*DCDC2*							
rs793704	*DCDC2*							
rs1087266	*DCDC2*							
rs2817220	*ALDH5A1*	1	0.0326	0.0067				0.0004
rs2252525	*ALDH5A1*							
rs807517	*ALDH5A1*	1		0.0225				0.0017
rs1054899	*ALDH5A1*							
rs2760184	Intergenic							
rs807527	*KIAA0319*							
rs699463	*KIAA0319*	1	0.0032	0.0231		0.0279	0.0153	0.0112
rs807530	*KIAA0319*							
rs807535	*KIAA0319*							
rs807540	*KIAA0319*	1	0.0204			0.0113	0.0093	
rs807545	*KIAA0319*	1					0.0244	
rs2817191	*KIAA0319*							
rs807521	*KIAA0319*	1					0.0306	
rs807525	*KIAA0319*							
rs807507	*KIAA0319*							
rs807508	*KIAA0319*	1					0.0304	

rs4504469	*KIAA0319*	1	0.0011	0.0082		0.004		0.01
rs4576240	*KIAA0319*							
rs4472344	*KIAA0319*							
rs16889511	*KIAA0319*							
rs4236032	*KIAA0319*	1				0.0432	0.0072	
rs9356939	*KIAA0319*	2	0.0156					0.0486
SNP24721800	*KIAA0319*	2				0.0205		
rs9393567	*KIAA0319*							
rs16889523	*KIAA0319*							
rs2745333	*KIAA0319*	1	0.0013	0.0073		0.0028		
rs12194307	*KIAA0319*							
rs2179515	*KIAA0319*	1	0.0012	0.0131		0.0004	0.0232	
rs730860	*KIAA0319*	2	0.0035		0.0132	0.0104		
rs761101	*KIAA0319*	1	0.0025	0.0057		0.0006	0.0325	
rs761100	*KIAA0319*	1	0.0027	0.0406	0.0107	0.0078	0.0306	
rs7766230	*KIAA0319*	2	0.0049		0.0002	0.0017	0.0096	
rs6935076	*KIAA0319*							
rs6456624	*KIAA0319*	1	0.0005	0.0045		0.0003	0.0157	
rs2328846	*KIAA0319*	1	0.0007	0.0017		0.0003	0.0155	
rs17491230[4]	*KIAA0319*	2	0.0023		0.0009	0.0041		
rs2038137	*KIAA0319*	1	0.0013	0.0026		0.0002	0.0061	
k_pr_del	Intergenic	1	0.0011	0.0032		0.0002	0.0086	
**rs3756821**	Intergenic	2		0.0401				
rs9467247[5]	Intergenic	2	0.0003	0.0362	0.0001	0.0002	0.002	
rs1555090	Intergenic	1	0.001	0.0029		0.0003	0.0131	
rs3212236[5]	Intergenic	2	0.0013		0.0006	0.0008	0.0024	
**rs13206167**	Intergenic							
**rs9461045**	Intergenic	2	0.0006	0.0489	0.0003	0.0003	0.0018	
**rs28501680**	Intergenic							
rs3033236	*TTRAP*	2	0.0134		0.0104	0.0073		
rs3212232	*TTRAP*							
rs2143340[5,6]	*TTRAP*	2	0.0063		0.0007	0.0016	0.0136	

[1] Markers new to this study are in bold
[2] 1 = major allele; 2 = minor allele
[3] Only nominally significant marker-trait associations ($P < 0.05$) are shown, and are uncorrected for multiple testing
[4] Equivalent to rs2235676 in Francks et al. [15]
[5] Re-genotyped in this study, resulting in different P-values than previously reported
[6] Risk haplotype-tagging SNP
