# Supplementary material for: A Common Variant Associated with Dyslexia Reduces Expression of the KIAA0319 Gene
Source: PLoS Genet. 2009 Mar 27;5(3):e1000436. doi: 10.1371/journal.pgen.1000436 (PMC2653637; doi:10.1371/journal.pgen.1000436)
Supplement: Text S1 — Supplementary methods. (0.07 MB PDF) [file pgen.1000436.s007.pdf]

Text S1. Supplementary methods

BAC recombineering

The primers below were used to amplify pGL3-Basic and add homology arms to both ends of the resulting PCR product. The 55-bp homologous region specific for the beginning or end of the targeted BAC sequence is in italics, and the sequence recognizing pGL3-Basic vector is underlined:

Beginning of *KIAA0319* upstream segment:
5'-*TAGGTGTTTAAATTTTGGCACTCCTGATATAAATAAGATTCTATTACTTCTAATT*C
GATAGAGAAATGTTCTGGCACCT-3'

End of *KIAA0319* upstream segment:
5'-*GATCCCCCGGGGGTGCAACCTTGCTCCACCTGTGCTGCCCTCGGCGGGCCTG*
*GCT*CGAGCTCTTACGCGTGCTA-3'.

Site-directed mutagenesis

The reverse primer for each oligonucleotide is the reverse complement of the forward oligonucleotide (unless otherwise included), and the mutant allele is underlined:

SNP 2_risk:
5'-aaaaaaccacttttaAtctgctttcatgagc-3'

SNP 4_risk:
5'-attatactaaggatacatcattttatccttGctacctcttgcagtgtctggc-3'
5'-aaggataaaatgatgtatccttagtataattaactttgtctgct-3'

SNP 5_risk:
5'-tgtctaaggctcttcTgtaaatacgagtagc-3'

ETF_mutant:
5'-ccgtggtaaccgcggAggcggaaaggcgctg-3'

RFX_mutant:
5'-cgtgcgcgcgcgtcgAgtggtaaccgcggcg-3'

Electrophoretic mobility shift assays

The following primers together with their reverse complements (not shown) were used to create double-stranded probe and competitor DNAs (with the exception of those purchased from Promega, which were already in double-stranded form):

SNP 2 non-risk: 5’-accacttttaGtctgctttca-3’

SNP 2 risk: 5’-accacttttaAtctgctttca-3'

SNP 4 non-risk: 5’-ttttatccttActacctcttg-3’

SNP 4 risk: 5’-ttttatccttGctacctcttg-3’

SNP 5 non-risk: 5’-aaggctcttcGgtaaatacgag-3'

SNP 5 risk: 5’-aaggctcttcTgtaaatacgag-3’

AP2: 5’-GATCGAACTGACCGCCCGCGGCCCGT-3’ (Promega)

OCT-1: 5’-TGTCGAATGCAAATCACTAGAA-3’ (Promega)

CRX: 5’-GGGGCGTTATTAATCATATTAATCCTCAC-3’

(CRX-binding site from the BAT-1 promoter [49])
